# Unveiling the role of gastric cancer-associated mesenchymal stem cells and neutrophil extracellular traps through multi-omics analysis

**DOI:** 10.1186/s13287-025-04768-7

**Published:** 2025-11-20

**Authors:** Zhengrui Li, Peng Luo, Zhaokai Zhou, Jijun Cao, Wen Zhang

**Affiliations:** 1 Shanghai Jiao Tong University School of Medicine, Shanghai, 200025 China; 2 Department of Oncology, Zhujiang Hospital, Southern Medical University, Guangzhou, 510280 Guangdong China; 3 Department of Urology, The First Affiliated Hospital of Zhengzhou University, Zhengzhou, 450052 Henan China; 4 Department of Urology, The Second Xiangya Hospital of Central South University, Changsha, Hunan, 410011 China; 5 Department of Laboratory Medicine, The First People’s Hospital of Taicang (Taicang Hospital Affiliated to Soochow University), No. 58, Changsheng South Road, Taicang, Suzhou, 215400 Jiangsu People’s Republic of China

**Keywords:** Gastric cancer, Tumor microenvironment, Mesenchymal stem cells, Neutrophil extracellular traps, Single-cell RNA sequencing, Mendelian randomization

## Abstract

**Background:**

Gastric cancer (GC) is a highly aggressive malignancy with a poor prognosis, closely linked to the tumor microenvironment (TME). Emerging evidence highlights the critical role of gastric cancer-associated mesenchymal stem cells (GC-MSCs) in recruiting neutrophils and facilitating neutrophil extracellular traps (NETs) formation, thereby remodeling the tumor microenvironment (TME) and promoting tumor progression, immune modulation, and metastasis.

**Methods:**

This study integrated single-cell RNA sequencing (scRNA-seq), Mendelian randomization (MR), and functional enrichment analyses to uncover the molecular underpinnings of GC. Transcriptomic data from public databases, including TCGA and GEO, were analyzed to explore cellular heterogeneity and the influence of NETs within the TME. MR analysis was conducted to establish causal relationships between key NET-related genes and GC. We integrated scRNA-seq, MR, and functional enrichment analyses to systematically dissect the roles of GC-MSCs and NETs in reshaping the tumor microenvironment.

**Results:**

scRNA-seq identified 12 distinct cell types in the TME, with significantly elevated NET scores associated with GC-MSCs in the disease group. Functional enrichment analyses revealed that NET-associated marker genes were enriched in pathways such as oxidative phosphorylation and cytoplasmic translation. MR analysis confirmed* EIF1* and *RPS12* as key genes with causal links to GC progression, demonstrating robust associations with immune cell infiltration and critical signaling pathways. Additionally, transcriptional regulation analysis identified motifs and transcription factors governing these genes, while GSEA highlighted their involvement in pathways such as ribosome biogenesis and focal adhesion.

**Conclusion:**

This study elucidates the intricate interplay between GC-MSCs, NETs, and the immune microenvironment, offering novel insights into GC pathogenesis and potential therapeutic targets. *EIF1 *and *RPS12* emerge as promising candidates for precision medicine, with broader implications for NET-related cancer research.

**Supplementary Information:**

The online version contains supplementary material available at 10.1186/s13287-025-04768-7.

## Introduction

Gastric cancer (GC) is a prevalent malignancy originating from the epithelial cells of the stomach. The incidence of GC is closely linked to a multitude of factors, including smoking, *Helicobacter pylori* (*H. pylori*) infection, dietary habits, environmental conditions, lifestyle choices, medication use, obesity, genetic predispositions, and other related variables. This cancer is characterized by its aggressive behavior, high propensity for metastasis, and poor prognosis [1, 2].

In recent years, the role of gastric cancer-associated mesenchymal stem cells (GC-MSCs) in tumor progression has garnered significant attention within the biomedical research community. MSCs, also referred to as stromal cells, are multipotent stem cells capable of differentiating into various cell types, including those of bone, adipose tissue, and cartilage. In the context of gastric cancer, MSCs are recruited to the tumor microenvironment (TME), where they have been demonstrated to play a pivotal role in tumor growth, angiogenesis, and immune evasion [3]. GC-MSCs have been implicated in promoting cancer invasion and metastasis through the secretion of various factors and the establishment of an immunosuppressive microenvironment [4]. Reports indicate that GC-MSCs can attract neutrophils to the TME, activate the STAT3 and ERK1/2 signaling pathways in neutrophils, and facilitate the metastasis of gastric cancer cells [5].

Neutrophils, as the frontline defenders of the immune system, are essential for the elimination of pathogens [6]. Recently, they have also been recognized as significant contributors to the TME, influencing cancer initiation and progression [7]. Furthermore, neutrophils release extracellular traps known as neutrophil extracellular traps (NETs), which are filamentous structures composed of DNA, histones, and proteins derived from cytotoxic granules [8]. These traps can induce thrombosis, leading to an increased incidence of cardiovascular events in cancer patients and ultimately contributing to mortality [9]. In addition, NETs facilitate the invasion, metastasis, and epithelial-mesenchymal transition (EMT) of tumor cells [10–12]. Understanding the complex interplay between NETs and tumor biology is crucial for elucidating the mechanisms underlying tumor progression and for the development of novel therapeutic strategies [13]. By targeting NETs or modulating their formation and function, it may be possible to disrupt their tumor-promoting effects and enhance the efficacy of existing cancer therapies.

In recent years, bioinformatics has emerged as a powerful tool in biomedical research, integrating molecular biology, genetics, statistics, and computer science to analyze and interpret biological data [14, 15]. This advanced technology not only deepens our understanding of diseases but also presents new opportunities for personalized treatment and early detection strategies. As advancements in bioinformatics continue, we can anticipate significant breakthroughs in the fight against gastric cancer [16].

Furthermore, within the realm of biomedical research, the ongoing progress in bioinformatics and genomics has highlighted Mendelian randomization (MR) as a robust method for investigating causal relationships between exposures and outcomes, particularly in clinical diseases or phenotypes [17, 18]. Based on the principle of random assignment of genetic variations at conception, MR ensures independence from confounding factors. This approach employs single nucleotide polymorphisms (SNPs) as instrumental variables to infer causality [18]. It is expected that MR will play a critical role in elucidating the causal pathways of various diseases, including gastric cancer [19]. For instance, researchers have utilized this method to explore the causal link between specific exposure factors, such as dietary habits or lifestyle choices, and the incidence of gastric cancer. This approach provides valuable insights into potential prevention strategies and therapeutic targets.

In recent years, single-cell RNA sequencing (scRNA-seq) has emerged as a transformative technology, enabling researchers to investigate individual cells with unprecedented resolution. By isolating and sequencing single cells, this approach facilitates the examination of genetic, epigenetic, and transcriptomic characteristics, thereby providing insights into cellular heterogeneity, interactions, and disease mechanisms that may remain obscured in traditional bulk sequencing methods. The primary advantage of scRNA-seq lies in its capacity to capture the diversity and dynamics of cell populations, revealing cell heterogeneity that is often masked in population-level analyses. This technology has significantly advanced our understanding of cellular development, tumor progression, and therapeutic responses [20, 21].In medical research, scRNA-seq has been widely applied; for example, in the context of gastric cancer, researchers have employed this methodology to analyze the TME, identify rare tumor subpopulations, and explore the molecular drivers of tumor progression. These findings offer valuable insights into the mechanisms underlying therapeutic resistance and highlight potential targets for personalized treatment [22]. As advancements in scRNA-seq technology continue, we anticipate substantial progress in elucidating the complex biology of gastric cancer and other diseases. The molecular-level analysis of individual cells holds the potential to transform diagnostic and therapeutic strategies, as well as enhance precision medicine in the fight against gastric cancer.

Building on these advancements, our study aims to further the understanding of GC by integrating scRNA-seq data with MR analysis to identify key genetic factors and cellular interactions that drive tumor progression. We hypothesize that specific GC-MSCs and their associated neutrophil interactions may serve as novel therapeutic targets. By dissecting the complex network of cellular crosstalk and genetic influences, we aim to uncover new pathways and biomarkers that could lead to improved prognostication and treatment strategies for GC patients. Our research will not only contribute to the fundamental understanding of GC pathogenesis but also pave the way for the development of precision medicine approaches tailored to individual patients’ genetic and cellular profiles.

## Materials and methods

### Study design and data acquisition

The present study employs a comprehensive approach, integrating transcriptomic data with genetic association studies to elucidate the molecular underpinnings of GC. We obtained expression profile data for 448 patients from The Cancer Genome Atlas (TCGA) public database, stratified into a control group of 36 and a disease group of 412. Additionally, single-cell RNA sequencing data from GSE183904 were procured from the NCBI Gene Expression Omnibus (GEO) database, comprising 36 samples with complete single-cell expression profiles. To explore the genetic architecture of blood gene expression, we utilized eQTL data from the eQTLGen Consortium, an initiative dedicated to understanding the genetic basis of complex traits through large-scale genome-wide meta-analysis in blood. Our study also leveraged summary statistics from the EBI database (ebi-a-GCST90018849), encompassing data from 1,029 GC cases and 475,087 controls, predominantly of European ancestry.

### Single-cell sequencing analysis

The single-cell sequencing data were processed using the ‘Seurat’ package in R, which facilitated the initial read-in of expression profiles and the subsequent exclusion of lowly expressed genes. We applied a standardized pipeline for data preprocessing, including quality control, normalization, principal component analysis (PCA), and Uniform Manifold Approximation and Projection (UMAP) to reduce dimensionality and visualize the data. The optimal number of principal components (PCs) was determined using ElbowPlot, and UMAP analysis was employed to discern the positional relationships between distinct cell clusters. Cluster annotation was performed using the Seurat package, focusing on cell types implicated in disease pathogenesis [23]. Marker genes for each cell subtype were extracted by setting the log fold change (LogFC) threshold parameter of the FindAllMarkers function to 0.25.

### GO and KEGG function enrichment analysis

To annotate the functions of differentially expressed genes and explore their functional correlations, we employed the R package “ClusterProfiler”. Gene Ontology (GO) and Kyoto Encyclopedia of Genes and Genomes (KEGG) pathways were utilized to assess relevant functional categories. Pathways with both p-value and q-value less than 0.05 were considered significantly enriched, providing insights into the biological processes and pathways associated with differentially expressed genes.

### Mendelian randomization analysis

Leveraging the extensive aggregated statistical data from the EBI database, we performed Mendelian randomization analysis to extract causal relationships in eQTL and identify single nucleotide polymorphisms (SNPs) as instrumental variables (IVs). A reliable MR analysis is based on three assumptions: (1) correlation hypothesis (instrumental variables are closely related to exposure but not directly related to outcomes), (2) independence hypothesis (instrumental variables cannot be related to confounding factors), and (3) exclusivity hypothesis (instrumental variables can only affect outcomes through exposure, and when IV can affect outcomes through other pathways, it is determined that there is genetic pleiotropy). SNPs associated with genes at the significance threshold (*P* < 1e-8) were selected, and linkage disequilibrium (LD) was calculated between them. SNPs with R^2^ < 0.001 and p < 1e-8 within a clustering window size of 10,000 kb were retained. We employed four statistical methods, Inverse variance weighted (IVW), MR Egger, Weighted median, and Weighted mode, to evaluate causal relationships. The reliability of these relationships was ascertained through a retention method, providing an overall estimate of the impact of gene expressions on GC risk.

### Sensitivity analysis and heterogeneity testing

To assess the impact of specific genetic variations on GC risk, we conducted Mendelian Randomization sensitivity analysis. This involved systematically excluding each SNP and recalculating the merged effect size of the remaining SNPs, generating new point estimates and 95% confidence intervals to evaluate their unique contributions and the robustness of the overall results. Heterogeneity testing was performed using the Mendelian heterogeneity test, calculating the weighted sum of squares of the effect size and standard error for each SNP to generate a Q value, which was then compared to a chi-square distribution.

### Immune cell infiltration analysis

The ‘CIBERSORT’ method, based on the principle of support vector regression, was used to evaluate immune cell types in microenvironments. This method contains 547 biomarkers and distinguishes 22 human immune cell phenotypes, including T cells, B cells, plasma cells, and myeloid cell subsets [24]. We utilized the ‘CIBERSORT’ algorithm to analyze patient data, inferring the relative proportion of 22 types of immune infiltrating cells and conducting Pearson correlation analysis on gene expression and immune cell content.

### GSEA pathway enrichment analysis

We further analyzed differences in signaling pathways between high and low expression groups through Gene Set Enrichment Analysis (GSEA). The background gene set was a version 7.0 annotated gene set downloaded from the MsigDB database, serving as the annotated gene set for subtype pathways. Differential expression analysis of pathways between subtypes was performed, and significantly enriched gene sets were sorted based on consistency scores (adjusted for p-values less than 0.05). GSEA is commonly used to explore the close combination of disease classification and biological significance.

### Gene set differential analysis (GSVA)

GSVA is a non-parametric and unsupervised method for evaluating the enrichment of transcriptome gene sets. GSVA converts gene level changes into pathway level changes by comprehensively scoring the set of genes of interest, thereby determining the biological function of the sample. We downloaded the hallmark gene set from the Molecular Signatures Database and used the GSVA algorithm to comprehensively score each gene set to evaluate potential biological functional changes in different samples.

### Transcriptional regulation analysis of key genes

This study predicts transcription factors using the R package “RcisTarget”. All calculations performed by RcisTarget are based on motif. The normalized enrichment score (NES) of motifs depends on the total number of motifs in the database. In addition to the motifs annotated by the source data, we also infer further annotation files based on motif similarity and gene sequence. The first step in estimating the overexpression of each motif on the gene set is to calculate the area under the curve (AUC) for each pair of motif motif sets. This is calculated based on the recovery curve of gene set for motif sorting. The NES of each motif is calculated based on the AUC distribution of all motifs in the gene set.

### Cell culture and siRNA transfection

The human gastric cancer cell line MKN45 was obtained from the Shanghai Jiao Tong University School of Medicine. Cells in the control group were cultured in DMEM medium (Gibco, USA) supplemented with 10% fetal bovine serum (FBS; Gibco, USA). All cell cultures were maintained in a humidified incubator at 37 °C with 5% CO₂.

Small interfering RNAs (siRNAs) targeting *EIF1* and *RPS12 *(two independent siRNAs for each gene, designated siRNA-1 and siRNA-2), as well as a negative control siRNA (si-NC), were synthesized by Tsingke Biotechnology Co., Ltd (Beijing, China). Transfections were carried out using Lipofectamine™ 2000 reagent (Invitrogen) according to the manufacturer’s protocol. Cells in the si-NC group were transfected with the negative control siRNA.

The siRNA sequences were as follows:

EIF1 siRNA-1: Sense: 5’-GGAACAGUUCUAUGAAUAA(dT)(dT)-3’, Antisense: 5’-UUAUUCAUAGAACUGUUCC(dT)(dT)-3’; EIF1 siRNA-2: Sense: 5’-GCUCAAAGAGGUUGAGAAA(dT)(dT)-3’, Antisense: 5′-UUUCUCAACCUCUUUGAGC(dT)(dT)-3′.

RPS12 siRNA-1: Sense: 5′-GGAUGGAGUUGAAAGUUAU(dT)(dT)-3′, Antisense: 5′-AUAACUUUCAACUCCAUCC(dT)(dT)-3′; RPS12 siRNA-2: Sense: 5′-CCAUGAUUCCAAAGAAUCA(dT)(dT)-3′, Antisense: 5′-UGAUUCUUUGGAAUCAUGG(dT)(dT)-3′.

### Colony formation assay

MKN45 cells and siRNA-transfected cells were seeded into 6-well plates at a density of 1000 cells per well and cultured for 5 days. The medium was refreshed every 2 days. At the end of the incubation period, cells were fixed with 4% paraformaldehyde and stained with 0.1% crystal violet for 30 min. Colonies were counted under a light microscope.

### Transwell migration assay

Cell migration was evaluated using Transwell chambers (Corning Inc., Corning, NY, USA). Briefly, 1 × 10^5^ cells suspended in serum-free medium were seeded into the upper chamber, while 500 µL of complete medium containing 10% FBS was added to the lower chamber as a chemoattractant. After incubation at 37 °C for 8 h in a 5% CO_2_ atmosphere, non-migrated cells on the upper surface of the membrane were carefully removed with cotton swabs. Migrated cells adherent to the lower surface were fixed with 4% paraformaldehyde for 15 min, stained with 0.1% crystal violet for 30 min, washed with distilled water, and counted under a light microscope in five randomly selected fields per insert.

### Quantitative real-time PCR (qRT-PCR)

Total RNA was extracted using TRIzol reagent and reverse-transcribed into complementary DNA (cDNA) using the Takara primescript RT reagent kit with gDNA eraser. qRT-PCR was performed using Roche FastStart Universal SYBR Green Master (ROX) reagents. The Roche Light Cycler480II qPCR instrument was adopted. Each sample was replicated in three Wells, and the results were analyzed using the 2^-ΔΔCT^ method. GAPDH served as the internal reference control.

Primer sequences were as follows: *GAPDH*: Forward: 5′-CTGGGCTACACTGAGCACC-3′, Reverse: 5′-AAGTGGTCGTTGAGGGCAATG-3′; *EIF1*: Forward: 5′-GAAACGGCAGGAAGACCCTTA-3′, Reverse: 5′-CGGATGCTCAATTACAGTACCAT-3′; *RPS12*: Forward: 5′-TGCTGGAGGTGTAATGGACG-3′, Reverse: 5′-GGCGCTTGTCTAAGGCTTTG-3′.

### Western blot analysis

Cells were harvested, washed with pre-chilled phosphate-buffered saline (PBS, pH 7.4), and lysed in RIPA buffer (Beyotime) supplemented with 1% phenylmethylsulfonyl fluoride (PMSF). Protein concentration was determined using the BCA Protein Assay Kit (Beyotime). Equal amounts of protein were resolved by sodium dodecyl sulfate-polyacrylamide gel electrophoresis (SDS-PAGE) and transferred to polyvinylidene fluoride (PVDF) membranes (Servicebio). After blocking for 1 h at room temperature, membranes were incubated overnight at 4 ℃ with the following primary antibodies: anti-EIF1 (1:1000, abs147590, absin, China), anti-RPS12 (1:2000, PS18100, abmart, China), and anti-GAPDH (1:5000; abs130609, absin, China ). After washing with TBST, membranes were incubated with secondary antibodies for 1 h at 37℃. Protein bands were visualized using an enhanced chemiluminescence (ECL) detection system.

### Statistical analysis

In this analysis, R Studio (version 4.2.2) was utilized. All statistical tests were two-tailed, and a p-value less than 0.05 was considered statistically significant(*, *p* < 0.05;**, *p* < 0.01; ***, *p* < 0.001).

## Results

### Single-cell sequencing analysis reveal gastric cancer fibroblast-associated NETs content

The expression profiles of the GSE183904 dataset from the GEO database were analyzed using the Seurat package. Cells were filtered based on the criteria, resulting in 145,494 cells retained for downstream analysis. The filtering process, visualized using violin plots (Supplementary Fig. 1A), minimized bias from low-quality data, ensuring high data integrity for subsequent analyses. The thresholds (nFeature_RNA > 500, nCount_RNA > 1000, percent.mt < 20, etc.) were chosen based on established practices in scRNA-seq preprocessing and confirmed by visual assessment of violin plots and scatterplots, as shown in *Supplementary Fig. 1A–C*.

Principal component analysis (PCA) was conducted to examine cellular similarities and differences, with points of varying colors representing distinct cell types (Supplementary Fig. 1B). This analysis provided essential insights for subsequent cell-type identification. The expression patterns of genes across cells were further analyzed, and the top 10 genes with the highest standardized variance were identified and visualized (Supplementary Fig. 1C). These genes exhibited significant differences in expression levels across cell types, highlighting their potential functional importance and reflecting the complexity and diversity of the cellular population.

Data normalization, PCA, and harmony analysis were performed (Supplementary Fig. 1D-F) to refine the single-cell data. These preprocessing steps enabled an in-depth exploration of gene expression, cell clustering, and individual differences, ensuring robust downstream analyses.

**Fig. 1:**
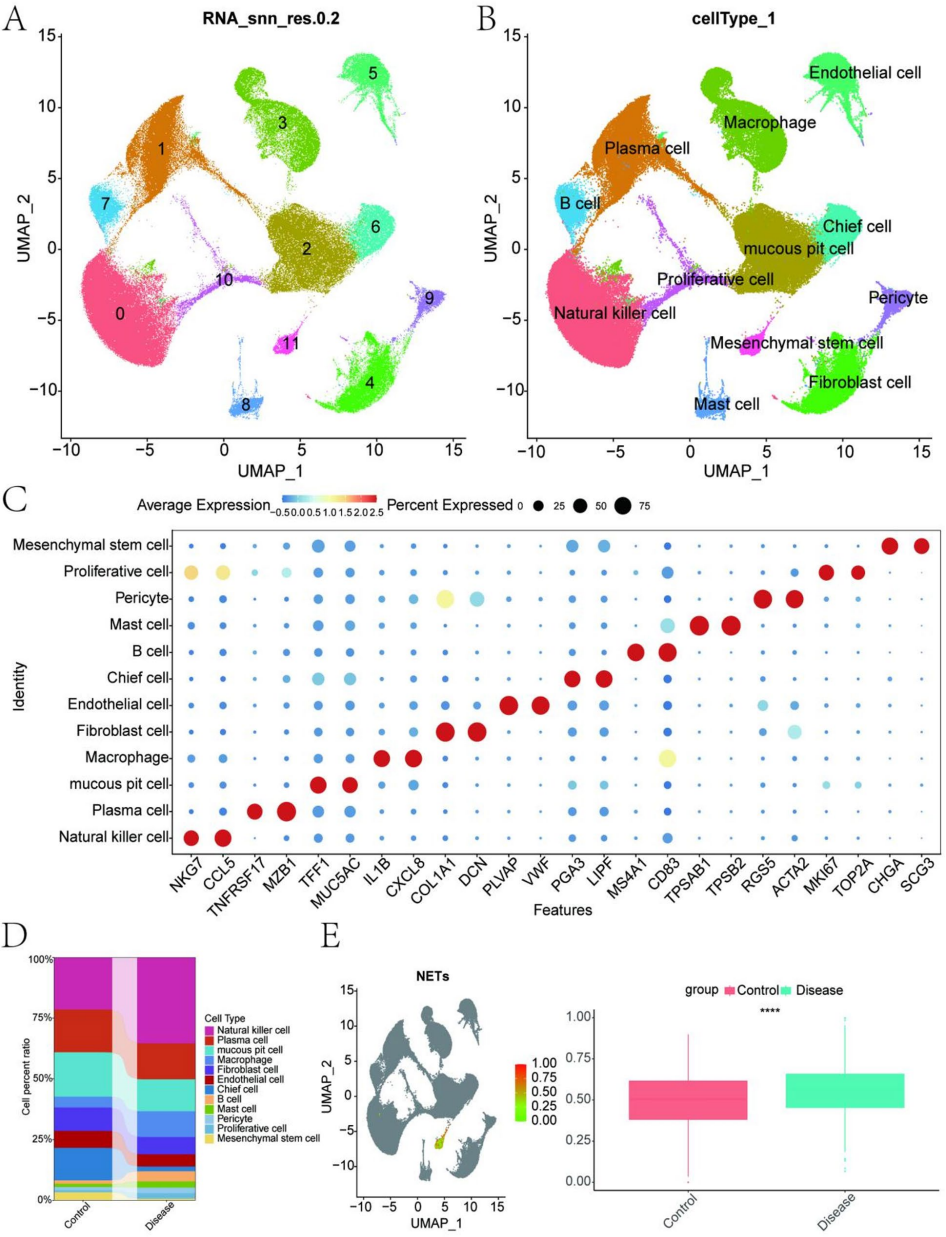
Cell clustering, annotation, and differential analysis of MSC-associated NETs scores. **A** UMAP visualization showing 12 cell clusters derived from PCA components, with each cluster represented by a distinct color. **B** Cell annotation results, identifying 12 cell types: Natural Killer (NK) Cells, Plasma Cells, Mucous Pit Cells, Macrophages, Fibroblast Cells, Endothelial Cells, Chief Cells, B Cells, Mast Cells, Pericytes, Proliferative Cells, and Mesenchymal Stem Cells (MSCs). **C** Bubble plot displaying the expression levels and percentages of marker genes across the 12 identified cell types. The size of each bubble represents the percentage of cells expressing the gene, while the color intensity reflects the average expression level. **D** Proportional differences of the 12 cell types between the control and disease groups, visualized as stacked bar plots. **E** Differential analysis of NETs scores evaluated at the single-cell level using the ssGSEA method. Left: UMAP plot showing the distribution of NETs scores across all cells. Right: Box plot comparing NETs scores between the control and disease groups, with the disease group showing a significantly higher score (****, *P* < 0.0001)

UMAP clustering based on RNA expression identified 12 distinct cell clusters, with positional relationships between clusters shown in Fig. 1A. Cell annotation classified these clusters into 12 cell types: Natural Killer (NK) Cells, Plasma Cells, Mucous Pit Cells, Macrophages, Fibroblast Cells, Endothelial Cells, Chief Cells, B Cells, Mast Cells, Pericytes, Proliferative Cells, and MSCs (Fig. 1B). A bubble plot of cell-type marker expression is presented in Fig. 1C. Figure 1D illustrates the differences in cell-type proportions between control and disease groups.

**Fig. 2:**
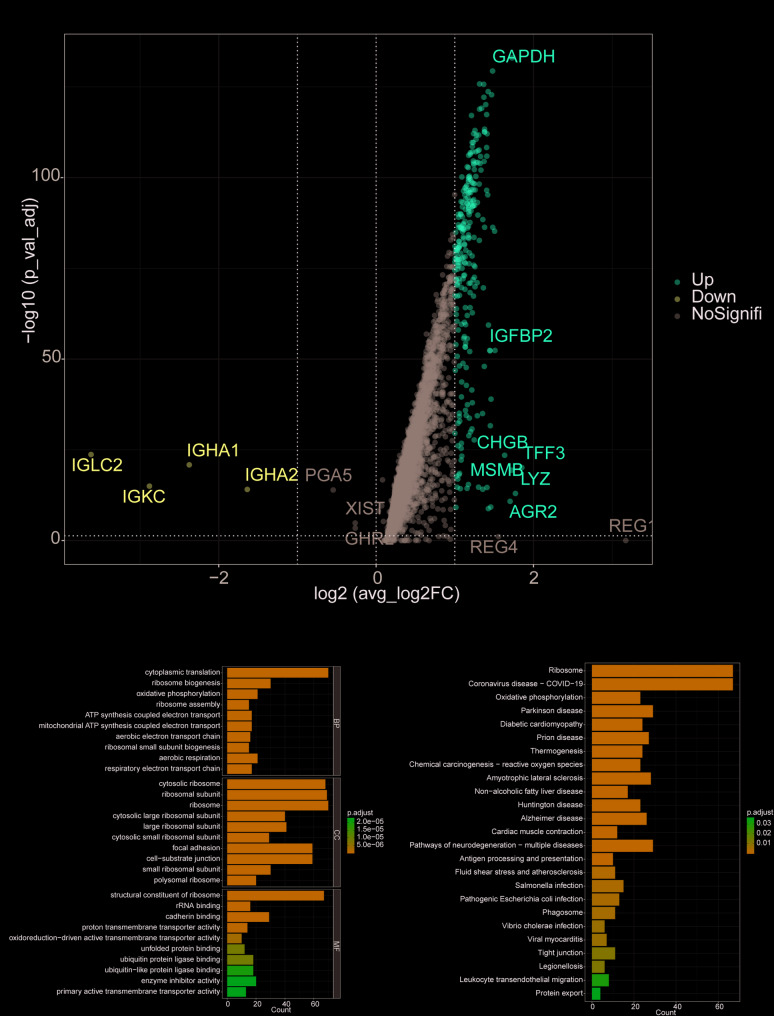
Differential expression and functional enrichment analysis of MSC-related NETs marker genes. **A** Volcano plot of differentially expressed genes (DEGs) associated with MSC-related NETs between the control and disease groups. Red dots represent upregulated DEGs, blue dots represent downregulated DEGs, and gray dots indicate genes without significant changes. **B** GO enrichment analysis of MSC-related NETs marker genes, highlighting pathways such as cytoplasmic translation, ribosome biogenesis, and oxidative phosphorylation. **C** KEGG enrichment analysis of MSC-related NETs marker genes, showing significant enrichment in pathways including Ribosome, Oxidative Phosphorylation, and Thermogenesis

At the single-cell level, NETs scores were evaluated using the ssGSEA function. The analysis revealed significantly higher Mesenchymal Stem Cell (MSC)-associated NETs scores in the disease group compared to the control group (Fig. 1E, ****P < 0.0001). The NETs gene set, derived from references [1, 2], included 214 NETs-associated genes. Differential analysis of high and low NETs score groups identified genes with significant differences, using the thresholds P-value < 0.05 and ∣avg log_2_FC∣>1. As shown in Fig. 2A, red points represent upregulated genes, blue points represent downregulated genes, and gray points represent genes with no significant change. In total, 269 differentially expressed genes were identified and visualized in a volcano plot (Fig. 2A).

### GO and KEGG functional annotation

To predict the biological functions of MSC-related marker genes, pathway enrichment analyses were conducted. GO enrichment analysis revealed that the intersecting genes were primarily enriched in pathways such as cytoplasmic translation, ribosome biogenesis, and oxidative phosphorylation (Fig. 2B). Similarly, KEGG enrichment analysis showed that these genes were significantly enriched in pathways including Ribosome, Oxidative Phosphorylation, and Thermogenesis (Fig. 2C).

### Mendelian randomization analysis

To identify key genes involved in gastric cancer influenced by MSC-associated NETs formation, we utilized the marker genes obtained from the previous analysis. Summary statistics from 476,116 samples (Controls: 475,087; Cases: 1,029) related to GC were retrieved, with the outcome ID ebi-a-GCST90018849. Using the extract_instruments and extract_outcome_data functions, 216 gene-outcome causal relationships were extracted.

**Fig. 3:**
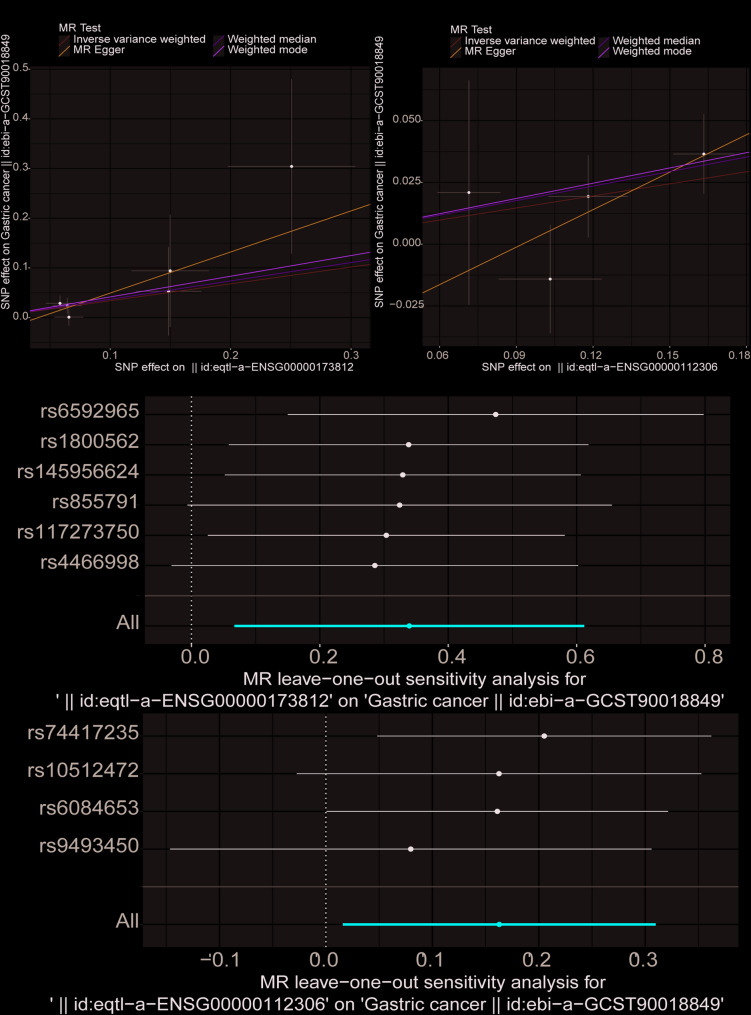
Mendelian Randomization and Sensitivity Analyses. **A** MR analysis showing the causal effect of EIF1 on GC. **B** MR analysis showing the causal effect of RPS12 on GC. **C** Leave-one-out sensitivity analysis demonstrating the robustness of EIF1’s causal effect on GC. **D** Leave-one-out sensitivity analysis demonstrating the robustness of RPS12’s causal effect on GC

MR analysis identified two genes, EIF1 and RPS12, with significant eQTL-positive causal relationships with GC (Fig. 3A-B, IVW *p* < 0.05). Specifically, EIF1 (OR: 1.404; CI: 1.069–1.844; *p* = 0.015) and RPS12 (OR: 1.177; CI: 1.016–1.363; *p* = 0.030) were associated with an increased risk of GC.

Sensitivity analysis using the leave-one-out method confirmed the robustness of these causal relationships. Results showed that removing any single SNP did not significantly impact the overall error line, supporting the stability of the causal associations (Fig. 3C-D). Further heterogeneity testing indicated that both EIF1 and RPS12 met the standards for heterogeneity, validating their reliability.

In summary, EIF1 and RPS12 were identified as critical genes linking MSC-associated NETs with GC development and progression, providing key targets for further investigation.

### Analysis of the relationship between RPS12, EIF1, and immune infiltration

The TME is a complex network composed of disease-associated fibroblasts, immune cells, extracellular matrix, various growth factors, inflammatory factors, unique physical and chemical characteristics, and cancer cells. The TME plays a critical role in disease diagnosis, prognosis, and clinical therapeutic sensitivity. 

**Fig. 4:**
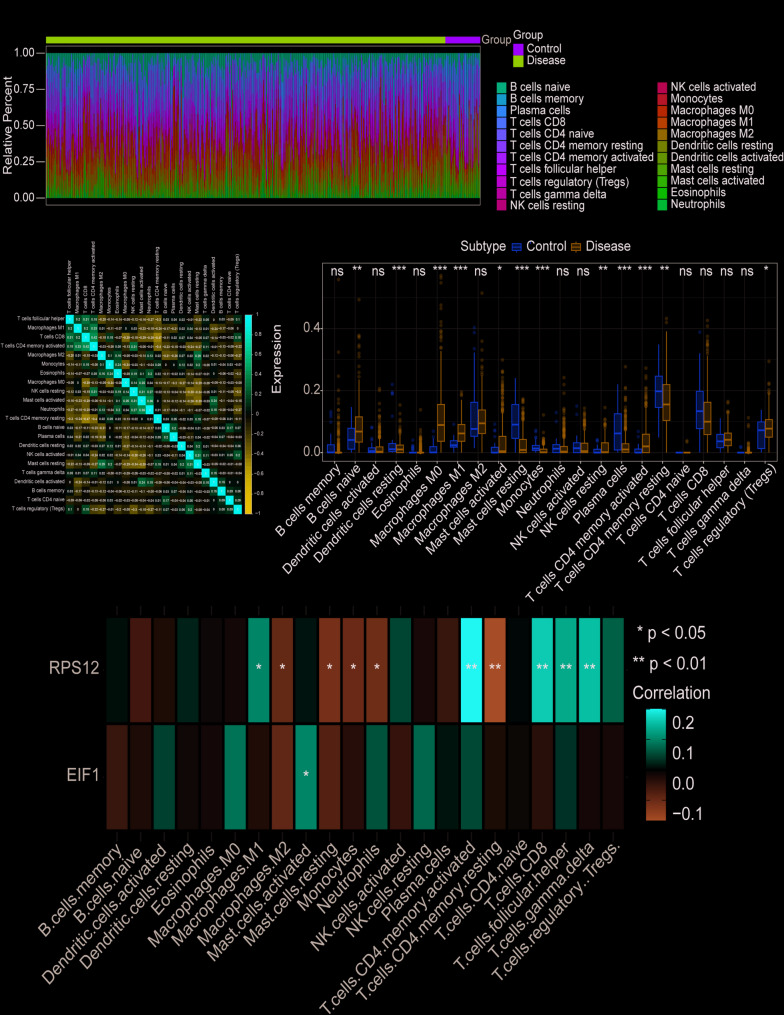
Immune infiltration analysis. **A** The relative proportions of 22 immune cell types in gastric cancer patients. **B** Pearson correlation matrix of the 22 immune cell types, with blue indicating negative correlations and red indicating positive correlations. **C** Box plots showing significant differences in immune cell contents between control and disease groups for selected cell types. **D** Correlation analysis of *RPS12* and *EIF1* with immune cells, with significant correlations marked (*, *P* < 0.05, **, *P* < 0.01)

To further explore the potential molecular mechanisms by which key genes influence gastric cancer progression, we analyzed their associations with immune infiltration in a gastric cancer dataset. The proportions of 22 types of immune cells across patients and their inter-cellular correlations were visualized (Fig. 4A-B). Significant differences were observed in the contents of immune cells such as B cells naive, Dendritic cells resting, Macrophages M0, Macrophages M1, Mast cells activated, Mast cells resting, Monocytes, NK cells resting, Plasma cells, T cells CD4 memory activated, T cells CD4 memory resting, and T cells regulatory (Tregs) between control and disease groups (Fig. 4C).

Further analysis revealed that *RPS12* was significantly positively correlated with T cells CD4 memory activated, T cells CD8, and T cells follicular helper, and negatively correlated with T cells CD4 memory resting, Macrophages M2, and Mast cells resting. Meanwhile, *EIF1* showed a significant positive correlation with Mast cells activated (Fig. 4D, *P < 0.05, **P < 0.01).

**Fig. 5:**
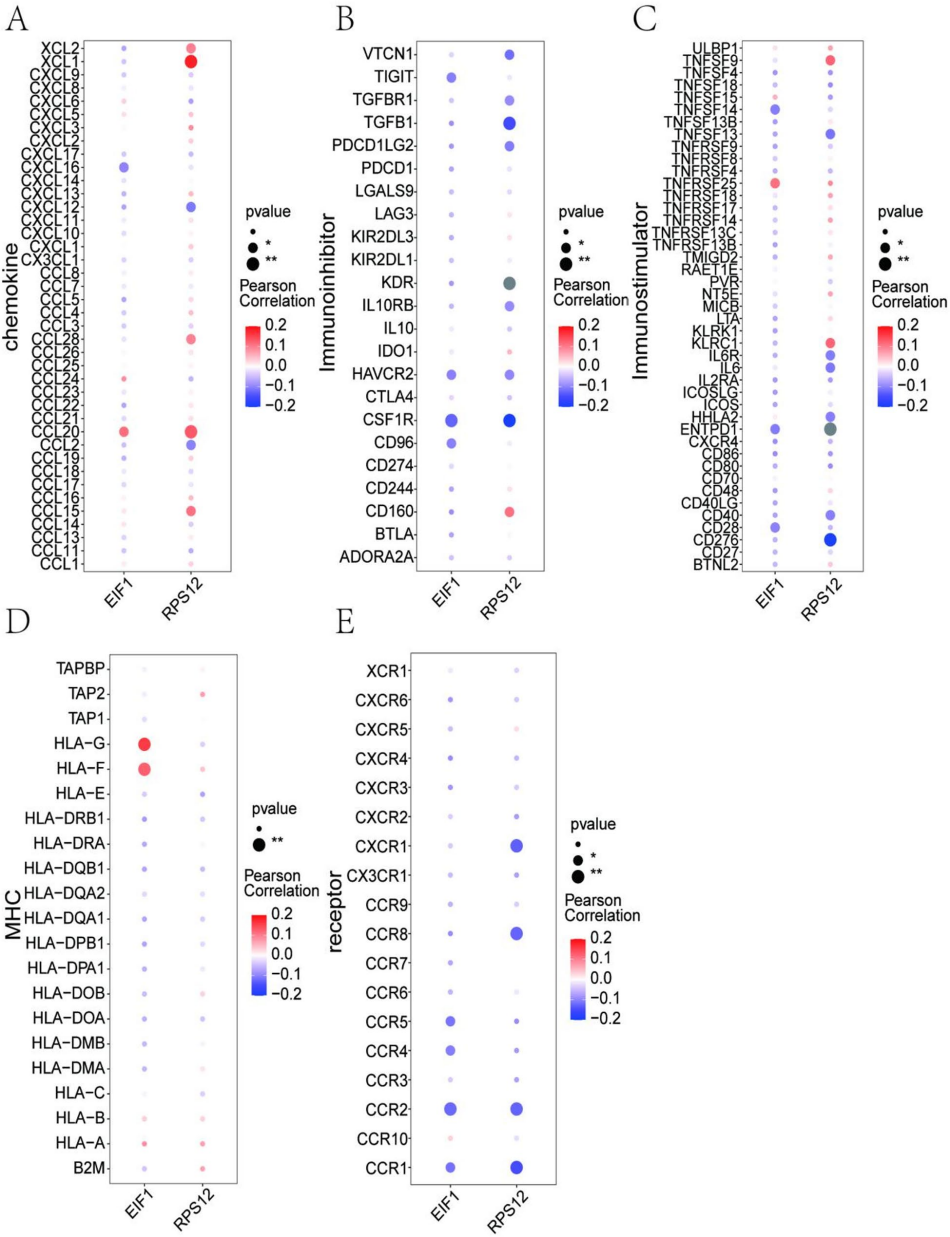
Relationships between key genes and immune factors. **A**-**E** Correlations between the key genes *EIF1* and *RPS12* with chemokines (**A**), immunoinhibitors (**B**), immunostimulators (**C**), MHC molecules (**D**), and receptors (**E**). The size of the dots represents the strength of the correlation, while the color intensity indicates the direction of the correlation

Additionally, correlations between key genes and immune factors, including immunosuppressive factors, immunostimulatory factors, chemokines, and receptors, were obtained from the TISIDB database. These findings suggest that the key genes *EIF1* and *RPS12* are closely related to immune cell infiltration and play a pivotal role in the immune microenvironment (Fig. 5).

### GSEA pathway enrichment analysis

**Fig. 6:**
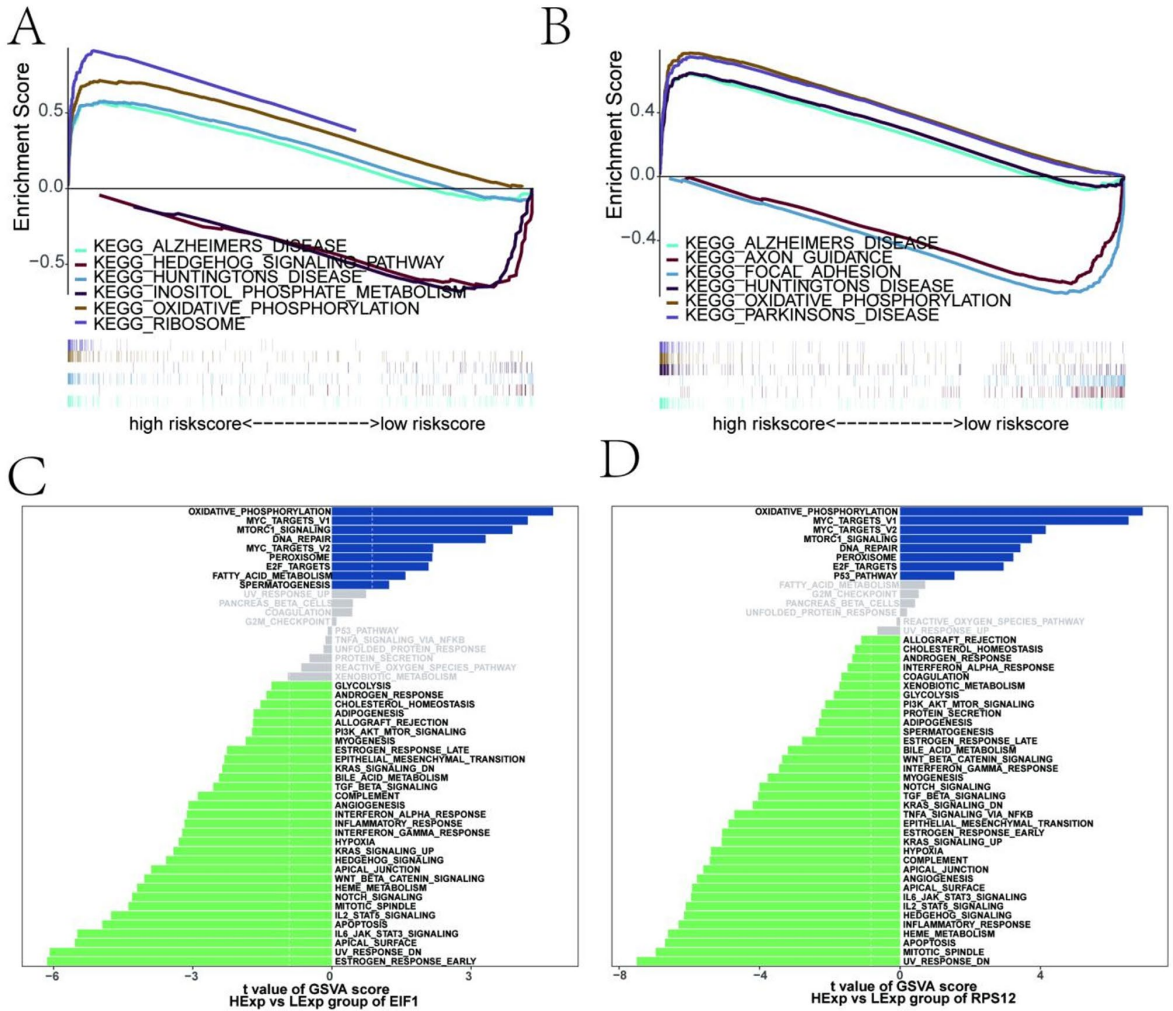
GSEA and GSVA pathway analysis of key genes. **A** GSVA database analysis of the key gene *EIF1*, showing its involvement in KEGG signaling pathways and the genes regulating these pathways. **B** GSVA database analysis of the key gene *RPS12*, showing its involvement in KEGG signaling pathways and the genes regulating these pathways. **C** GSEA results for *EIF1*, demonstrating significant enrichment in pathways such as KEGG_RIBOSOME, KEGG_HEDGEHOG_SIGNALING_PATHWAY, and KEGG_OXIDATIVE_PHOSPHORYLATION. **D** GSEA results for *RPS12*, demonstrating significant enrichment in pathways such as KEGG_AXON_GUIDANCE, KEGG_FOCAL_ADHESION, and KEGG_OXIDATIVE_PHOSPHORYLATION. In the GSVA analysis, blue bars represent pathways enriched in the high-expression group, while green bars represent pathways enriched in the low-expression group

To investigate the molecular mechanisms through which key genes influence gastric cancer progression, GSEA was performed to compare differences in signaling pathways between high and low-expression groups. Additionally, the GSVA database was utilized to explore the specific pathways involving the key genes *EIF1* and *RPS12* and the regulatory genes associated with these pathways (Fig. 6A-B).

GSEA results revealed that *EIF1* was significantly enriched in pathways such as KEGG_RIBOSOME, KEGG_HEDGEHOG_SIGNALING_PATHWAY, and KEGG_OXIDATIVE_PHOSPHORYLATION (Fig. 6C). Similarly, *RPS12* was enriched in pathways including KEGG_AXON_GUIDANCE, KEGG_FOCAL_ADHESION, and KEGG_OXIDATIVE_PHOSPHORYLATION (Fig. 6D). These findings highlight the roles of *EIF1* and *RPS12* in crucial signaling pathways, potentially contributing to the progression and regulation of gastric cancer.

### Transcriptional regulation analysis of key genes

**Fig. 7:**
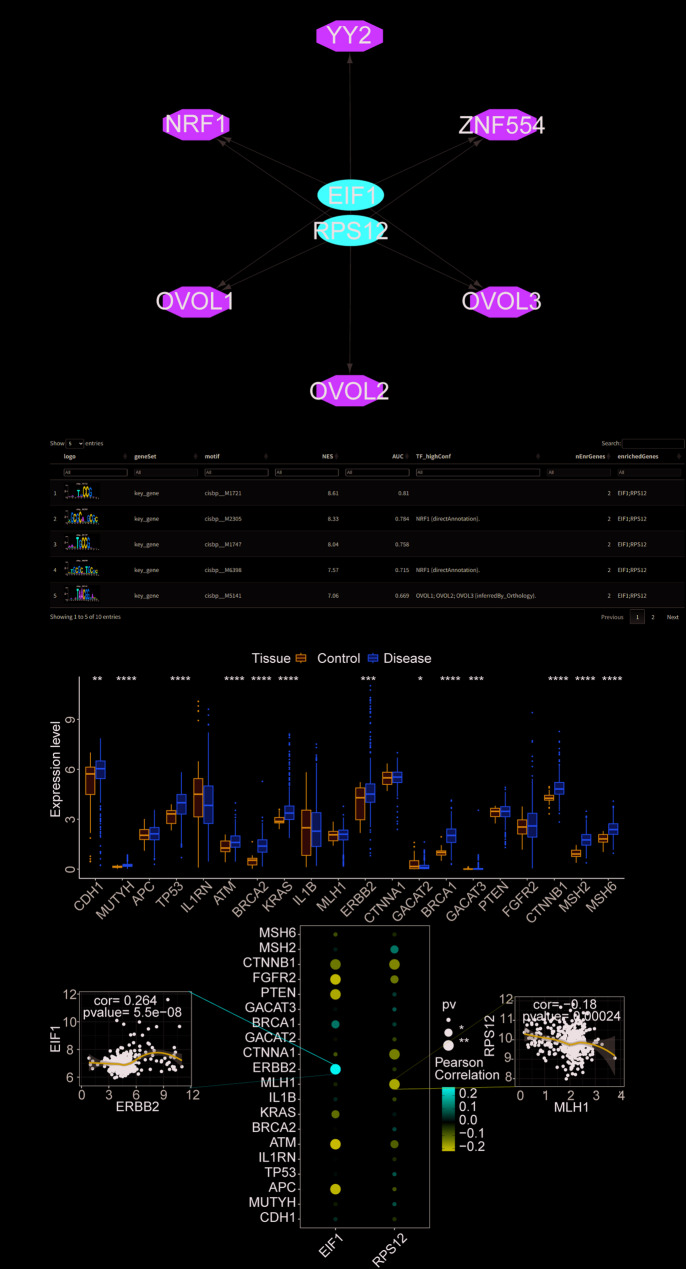
Transcriptional regulation and disease correlation of key genes. **A** Transcriptional regulatory network of key genes, where red represents key genes (EIF1 and RPS12) and green represents transcription factors regulating these genes. **B** Display of enriched motifs and their corresponding transcription factors for key genes. **C** The upper panel illustrates the differential expression of disease-regulating genes between control (blue) and disease (yellow) patients. The lower panel shows the Pearson correlation analysis of key genes with disease-regulating genes. Blue indicates a negative correlation, and red indicates a positive correlation

The two key genes (*EIF1* and *RPS12*) were analyzed as part of the current study’s gene set, revealing that they are regulated by multiple transcription factors (TFs) through shared mechanisms. Enrichment analysis of these TFs was performed using cumulative recovery curves. Motif-TF annotation and key gene selection analysis showed that the motif cisbp__M1721 had the highest normalized enrichment score (NES: 8.61). All motifs enriched for key genes and their corresponding TFs are presented (Fig. 7A-B).

Using the GeneCards database (https://www.genecards.org/), gastric cancer-related disease-regulating genes were identified. Differential expression analysis showed that genes such as *CDH1, MUTYH, TP53, ATM, BRCA2, KRAS, ERBB2, GACAT2, BRCA1, GACAT3, CTNNB1, MSH2*, and *MSH6* had significant expression differences between control and disease groups. Further correlation analysis revealed significant associations between the expression levels of key genes and those of disease-related genes. Notably, EIF1 was positively correlated with (cor = 0.264), and *RPS12* was negatively correlated with *MLH1* (cor=-0.18) (Fig. 7C).

**Fig. 8:**
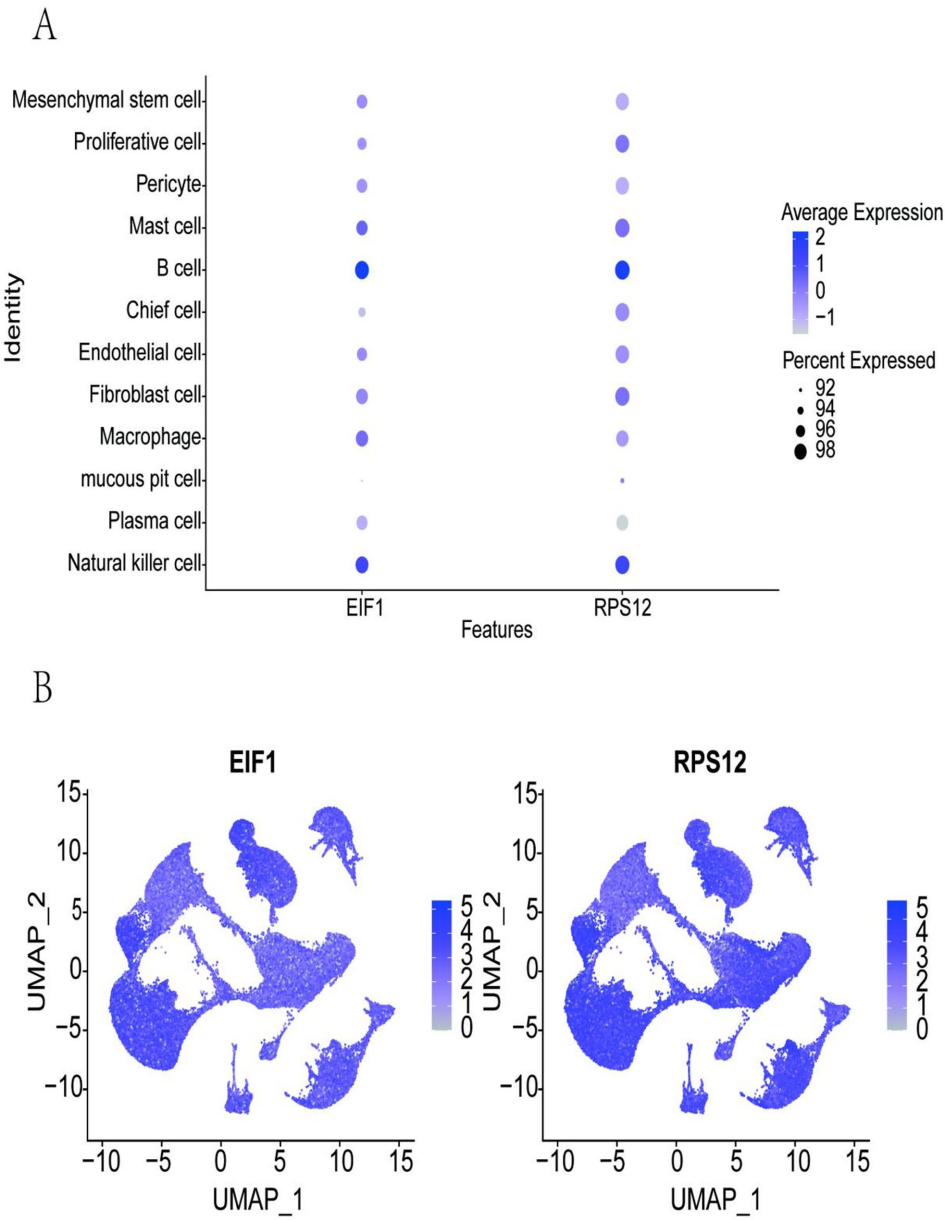
Expression of key genes in single cells. **A** Expression of *EIF1* and *RPS12* in various single-cell types. **B** Distribution of key genes across 12 cell types, including Natural killer cells, Plasma cells, Mucous pit cells, Macrophages, Fibroblast cells, Endothelial cells, Chief cells, B cells, Mast cells, Pericytes, Proliferative cells, and MSCs

Single-cell data analysis showed the expression profiles of key genes across various cell types, including MSCs, Proliferative cells, Pericytes, Mast cells, B cells, Chief cells, Endothelial cells, Fibroblast cells, Macrophages, Mucous pit cells, Plasma cells, and Natural killer cells (Fig. 8A-B). Additionally, co-expression analysis of key genes with disease-regulating genes in single-cell data was conducted, highlighting the top 5 co-expressed genes (Supplementary Fig. 2, Supplementary Fig. 3).

#### Validation of *EIF1* and *RPS12* in GC cell line

**Fig. 9:**
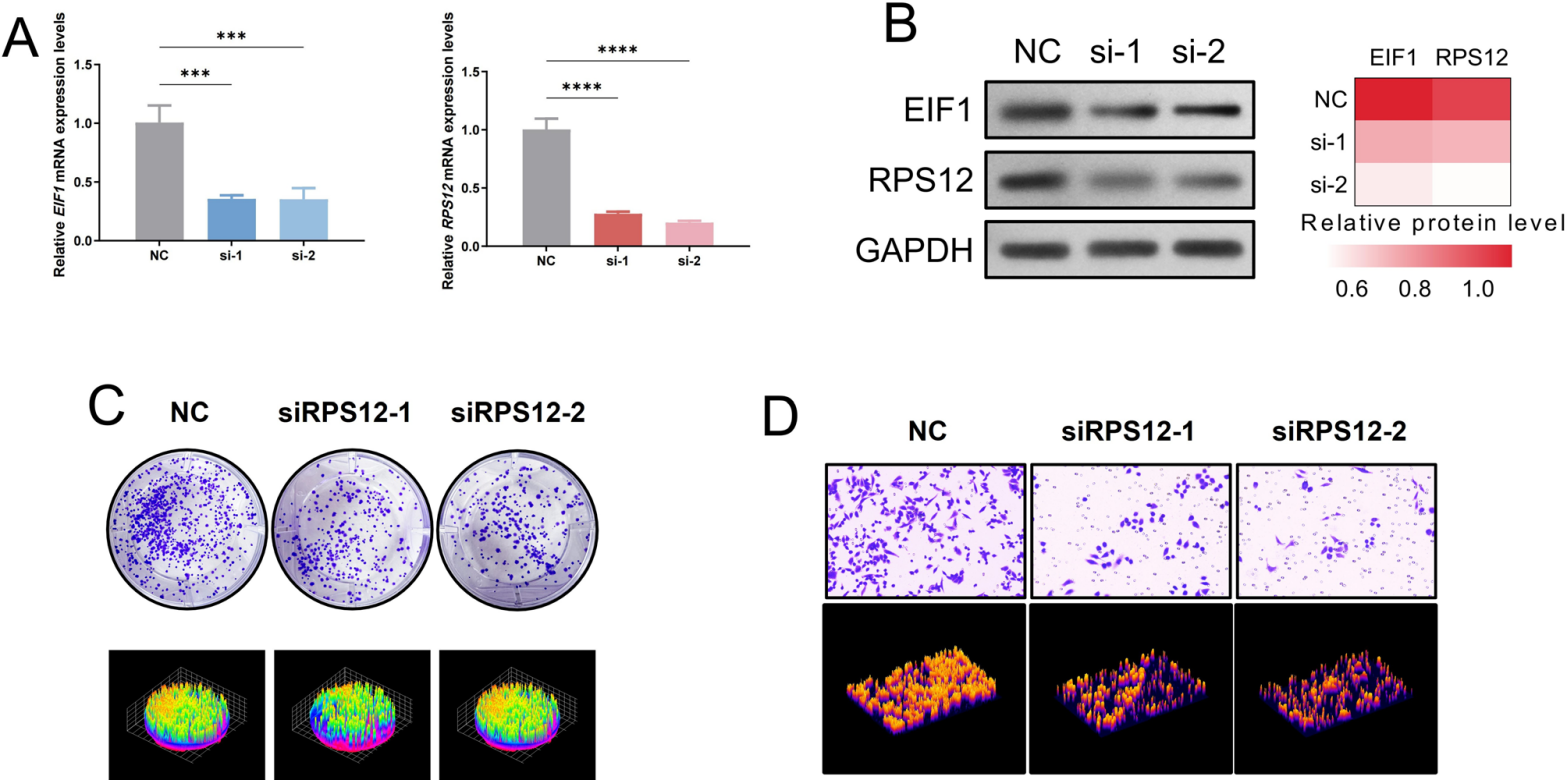
Functional validation of EIF1 and RPS12 knockdown in gastric cancer cells (MKN45). **A** Quantitative real-time PCR analysis showing significantly reduced EIF1 (left) and RPS12 (right) mRNA expression after siRNA-mediated knockdown with two independent siRNAs (si-1, si-2) compared to negative control (NC). Data are presented as mean ± SD, ***P < 0.001, ****P < 0.0001. **B** Western blot analysis confirming decreased protein levels of EIF1 and RPS12 after knockdown, with GAPDH as loading control. Relative protein quantification is shown as a heatmap. **C** Colony formation assays demonstrating reduced number and size of colonies following RPS12 knockdown compared with NC. Representative images (top) and semi-quantitative 3D rendering of colony density (bottom) are shown. **D** Transwell migration assays showing significantly fewer migrated cells upon RPS12 knockdown compared to NC. Representative microscopic images (top) and 3D rendering of stained cell density (bottom) are shown

To experimentally validate our computational findings, we silenced *EIF1* and *RPS12* using two independent siRNAs for each gene (si-1 and si-2) in GC cell line MKN45. RT-qPCR confirmed that both *EIF1* and *RPS12* mRNA levels were significantly reduced compared to the negative control (NC) (Fig. 9A). Western blot analysis further verified effective knockdown at the protein level, with relative quantification shown as a heatmap (Fig. 9B). Plate clone formation assay demonstrated that silencing *EIF1* or *RPS12* markedly reduced colony number and size compared with NC (Fig. 9C). Quantitative analysis confirmed significant impairment of proliferative capacity in both knockdown groups. Transwell assays revealed that knockdown of *EIF1* or *RPS12* significantly decreased the number of migrated cells relative to NC (Fig. 9D). Semi-quantitative 3D rendering of stained cells further emphasized the substantial reduction in migratory potential following gene silencing.

## Discussion

This study systematically uncovers the complex interactions between GC-MSCs and neutrophil extracellular traps through multi-omics technologies. It provides a fresh perspective on tumor microenvironment research. Unlike traditional approaches that focus on single molecular mechanisms or signaling pathways, this study integrates single-cell RNA sequencing with Mendelian randomization analysis, offering a multi-layered understanding of the critical role of MSC-related NETs in gastric cancer [25, 26] .

Single-cell sequencing captures cellular heterogeneity and the functional characteristics of various cell subpopulations within the TME, avoiding the loss of biological information due to heterogeneity dilution in traditional bulk RNA sequencing [27]. The findings reveal that MSC-related NET scores are significantly elevated in GC patients compared to healthy controls. Functional enrichment analysis further demonstrates that associated marker genes are enriched in key biological pathways such as cytoplasmic translation and oxidative phosphorylation. These discoveries not only deepen our understanding of the roles of MSCs and NETs in GC progression but also provide novel insights into the molecular mechanisms underlying GC [28, 29].

Importantly, our MR analysis validated the causal relationships between *EIF1* and *RPS12 *and GC development, providing robust genetic evidence for their involvement in tumor biology. We further complemented these in silico findings with in vitro knockdown experiments. Silencing *EIF1* or *RPS12* significantly reduced their mRNA and protein expression, inhibited colony formation, and impaired cell migration, thereby supporting their biological significance and potential as therapeutic targets. These functional assays highlight that *EIF1* and *RPS12* are not merely associated markers but active drivers of GC progression.

Mechanistically, *EIF1 *and *RPS12* are central regulators of ribosome biogenesis and translational initiation, processes that are frequently upregulated in cancer cells. By enhancing ribosome production and protein synthesis, these genes facilitate rapid cell proliferation and stress adaptation. In parallel, the enrichment of oxidative phosphorylation pathways links these genes to mitochondrial metabolism and reactive oxygen species (ROS) production. Elevated ROS levels are well-known triggers of NET formation, thereby providing a plausible mechanistic bridge between tumor-intrinsic translational/metabolic reprogramming and NET-associated immune modulation in GC. In this context, *EIF1* and *RPS12* may serve as molecular hubs connecting metabolic and translational machinery with immune regulation, ultimately promoting a tumor-permissive microenvironment.

Moreover, the functions of these genes are not static; their roles may dynamically change with tumor progression or therapeutic intervention. Future studies should employ long-term dynamic sampling with time-series scRNA-seq to monitor the expression patterns of *EIF1* and *RPS12*. Additionally, spatial transcriptomics can be utilized to determine their specific distribution within tumor tissues, further elucidating their bridging roles between tumor stroma and immune cells and providing new evidence for their molecular functions.

Beyond GC, the universal relevance of NET formation mechanisms and their marker genes across other cancer types warrants attention [30]. For instance, analyzing public datasets of cancers such as pancreatic, lung, and liver cancers may reveal the potential of *EIF1* and *RPS12* as broad-spectrum anti-tumor targets [31]. Such cross-cancer investigations can expand the applicability of *EIF1* and *RPS12* and offer a comprehensive understanding of the general mechanisms of NETs in tumor progression [32, 33]. Furthermore, given the dynamic and complex nature of NET formation, which involves coordinated regulation across multiple pathways, including metabolic reprogramming, immune evasion, and inflammatory signaling, future research could integrate metabolomics and epigenomics to dissect the regulatory networks of *EIF1* and *RPS12*. This would further reveal their potential roles in chemotherapy and immunotherapy resistance.

Despite the significant findings, this study has certain limitations. First, the scRNA-seq data used in this study are relatively limited in sample size, relying solely on a single dataset from a public database, which may not fully capture the biological heterogeneity of GC patients [22, 34, 35]. Second, the MR analysis results are primarily based on genetic data from European populations, which may not generalize to other ethnic groups or regions. Future studies should include larger, multi-center cohorts with diverse populations to ensure the generalizability of the conclusions. Moreover, while this study proposes the therapeutic potential of *EIF1* and *RPS12 *as targets, this hypothesis requires further validation through in-depth in vitro and in vivo experiments, such as functional testing in patient-derived organoids and therapeutic evaluation in animal models.

In MR analysis, horizontal pleiotropy poses a risk to causal inference. To mitigate this, we applied heterogeneity tests and MR-Egger regression. SNPs were filtered based on LD (R² < 0.001) and genome-wide significance (*P* < 1e-8), ensuring independence from confounders. Leave-one-out sensitivity analysis further validated the robustness of our results.

Clinically, *EIF1* and *RPS12* may serve as biomarkers for patient stratification or therapeutic response. Their involvement in immune regulation and translational machinery suggests potential for targeted therapy. Drug screening and gene-editing technologies could be leveraged to evaluate these targets in preclinical models.

The scRNA-seq dataset was derived from a single public source, which may introduce sampling bias and limit generalizability. Moreover, the MR dataset was based on European populations, which may not capture population-specific genetic architectures. These factors may affect the reproducibility and translatability of our findings.

In conclusion, this study reveals the molecular and cellular mechanisms underlying the interaction between MSC-related NETs and GC, offering a novel perspective for understanding the complexity of GC progression. Scientifically, these findings provide potential targets for early diagnosis and precision therapy of GC, with broader implications for NET-related disease research. Future studies should integrate multi-omics data, including proteomics, metabolomics, and spatial transcriptomics, to comprehensively elucidate the roles of NETs in GC and other cancers. Additionally, based on the results of this study, gene-editing tools or targeted drugs for *EIF1* and *RPS12* could be developed and evaluated through multi-center clinical trials for their potential in precision medicine. By bridging basic research with clinical applications, this field holds promise for developing more effective personalized treatment strategies to benefit a broader range of patients.

## Conclusion

This study integrates single-cell RNA sequencing, Mendelian randomization, and multi-omics analyses to elucidate the intricate interplay between gastric cancer-associated GC-MSCs and NETs in the tumor microenvironment. Key findings include the identification of *EIF1* and *RPS12* as critical genes with causal links to gastric cancer progression, significantly influencing immune infiltration and key signaling pathways such as oxidative phosphorylation and ribosome biogenesis. These results highlight the dynamic role of NETs in promoting tumor progression and immune modulation, offering novel insights into gastric cancer pathogenesis and potential therapeutic targets. Future research should expand on these findings by incorporating larger, multi-center cohorts, in-depth functional experiments, and multi-omics data to validate and translate these discoveries into precision medicine strategies.

## Supplementary Information

Below is the link to the electronic supplementary material.

Supplementary Material 1.

## Abbreviations

GC: Gastric Cancer
TME: Tumor Microenvironment
GC-MSCs: Gastric Cancer-Associated Mesenchymal Stem Cells
NETs: Neutrophil Extracellular Traps
scRNA-seq: Single-Cell RNA Sequencing
MR: Mendelian Randomization
TCGA: The Cancer Genome Atlas
GEO: Gene Expression Omnibus
eQTL: Expression Quantitative Trait Loci
SNPs: Single Nucleotide Polymorphisms
PCA: Principal Component Analysis
UMAP: Uniform Manifold Approximation and Projection
GO: Gene Ontology
KEGG: Kyoto Encyclopedia of Genes and Genomes
IVW: Inverse Variance Weighted
GSVA: Gene Set Variation Analysis
GSEA: Gene Set Enrichment Analysis
NES: Normalized Enrichment Score
IVs: Instrumental Variables
CI: Confidence Interval
OR: Odds Ratio
TFs: Transcription Factors
BRCA: Breast Cancer Gene
ERBB2: Erb-B2 Receptor Tyrosine Kinase 2
CXCR4: C-X-C Chemokine Receptor Type 4
MHC: Major Histocompatibility Complex
