# Supplementary material for: Unveiling the role of gastric cancer-associated mesenchymal stem cells and neutrophil extracellular traps through multi-omics analysis
Source: Stem Cell Res Ther. 2025 Nov 20;16:651. doi: 10.1186/s13287-025-04768-7 (PMC12632061; doi:10.1186/s13287-025-04768-7)
Supplement: Supplementary file 1 — Supplementary Material 1. [file 13287_2025_4768_MOESM1_ESM.docx]

**Supplementary Figures**


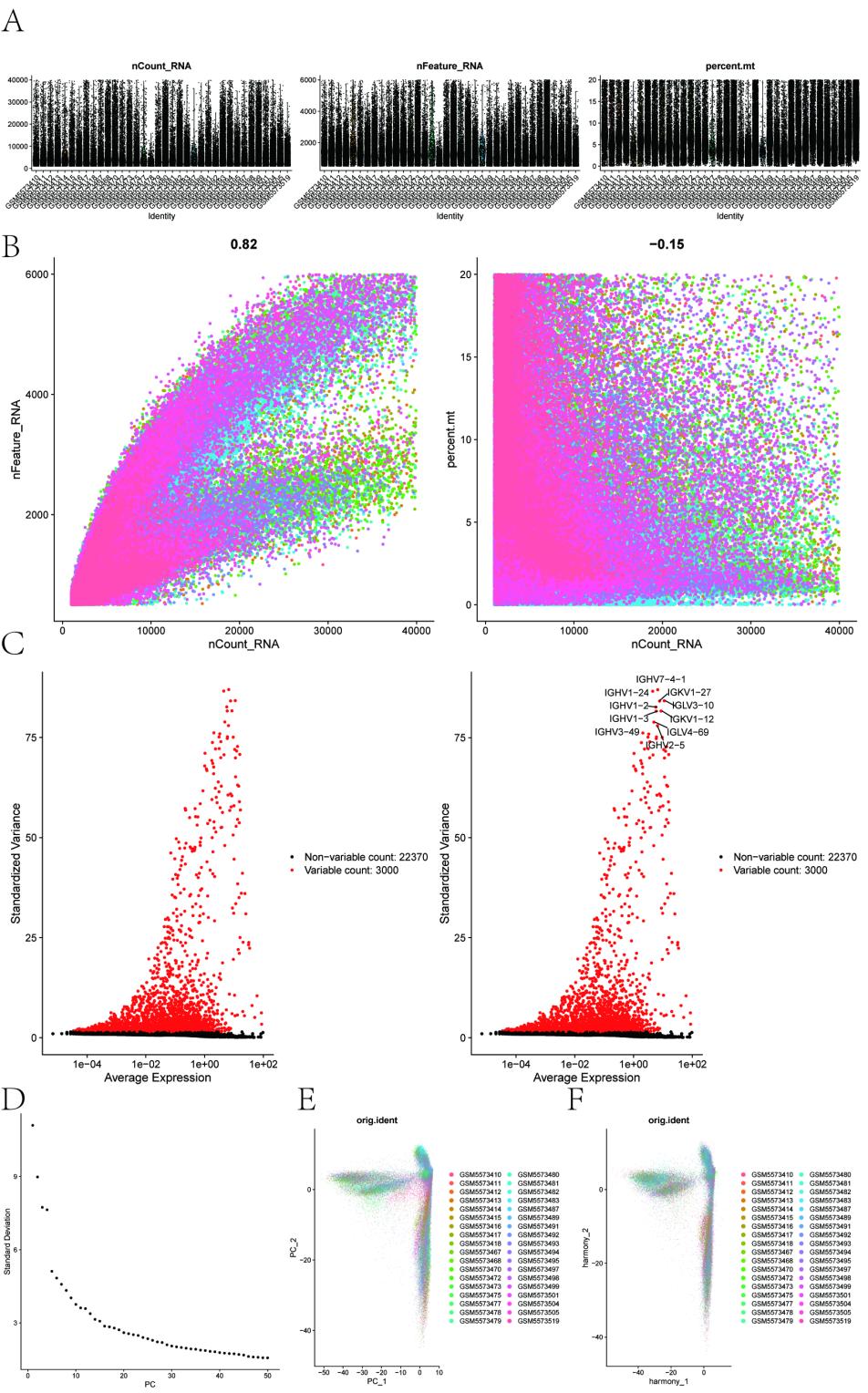


S.1. Quality control and PCA analysis of single-cell datasets. (A) the total number of “nCount_RNA ” and “nFeature_RNA” and the “percent.mt” of each sample. (B) the correlation between “nCount_RNA” and “nFeature_RNA” and “percent.mt”. (C) the top 3000 highly variable genes are marked in red. (D) The variance ranking diagram of each PC. 20 PCs with P < 0.05 were identified using the JackStraw function.(E-F) The display and the distribution of PC. the point represents the cell, and the color represents the sample.


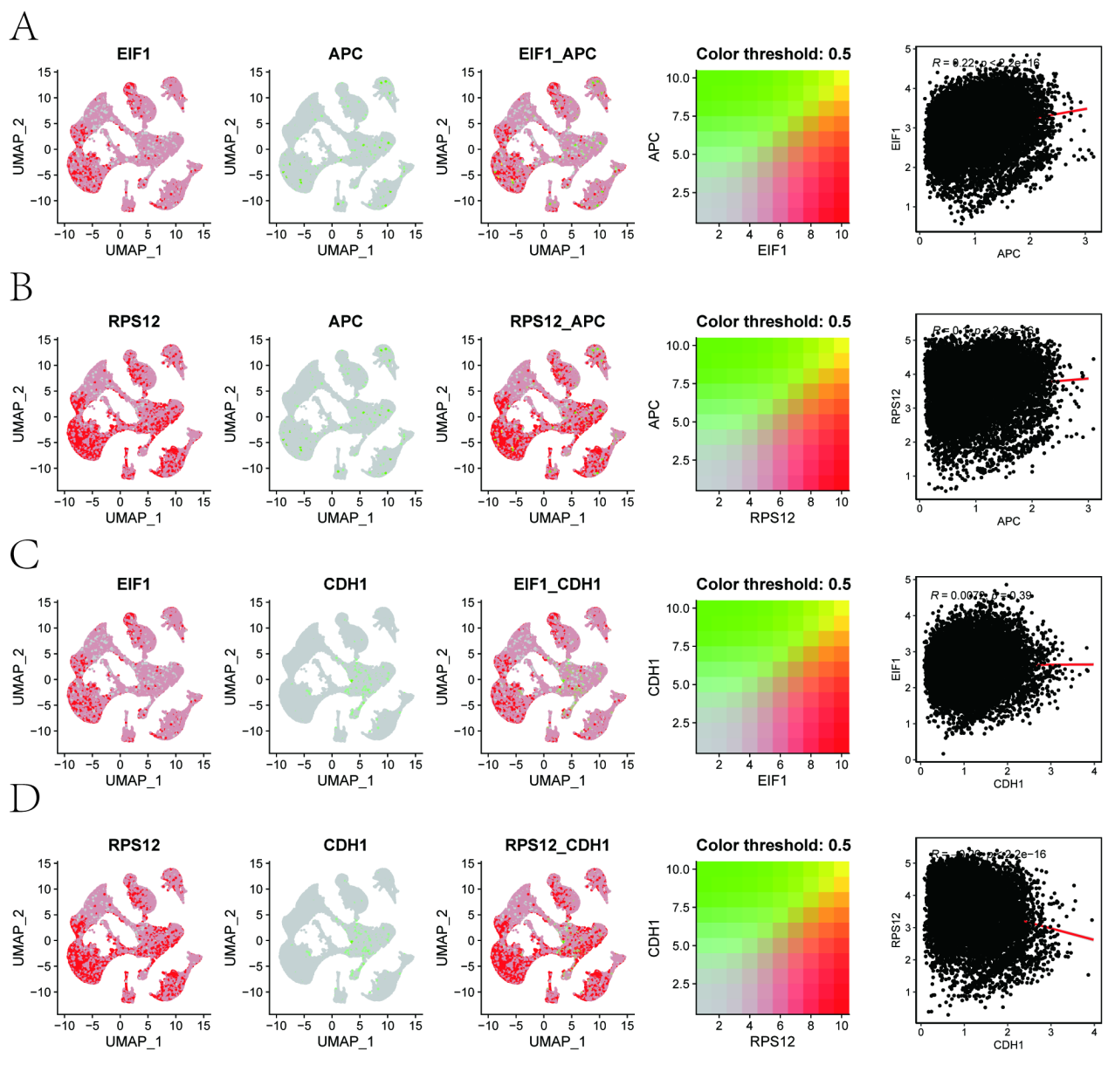


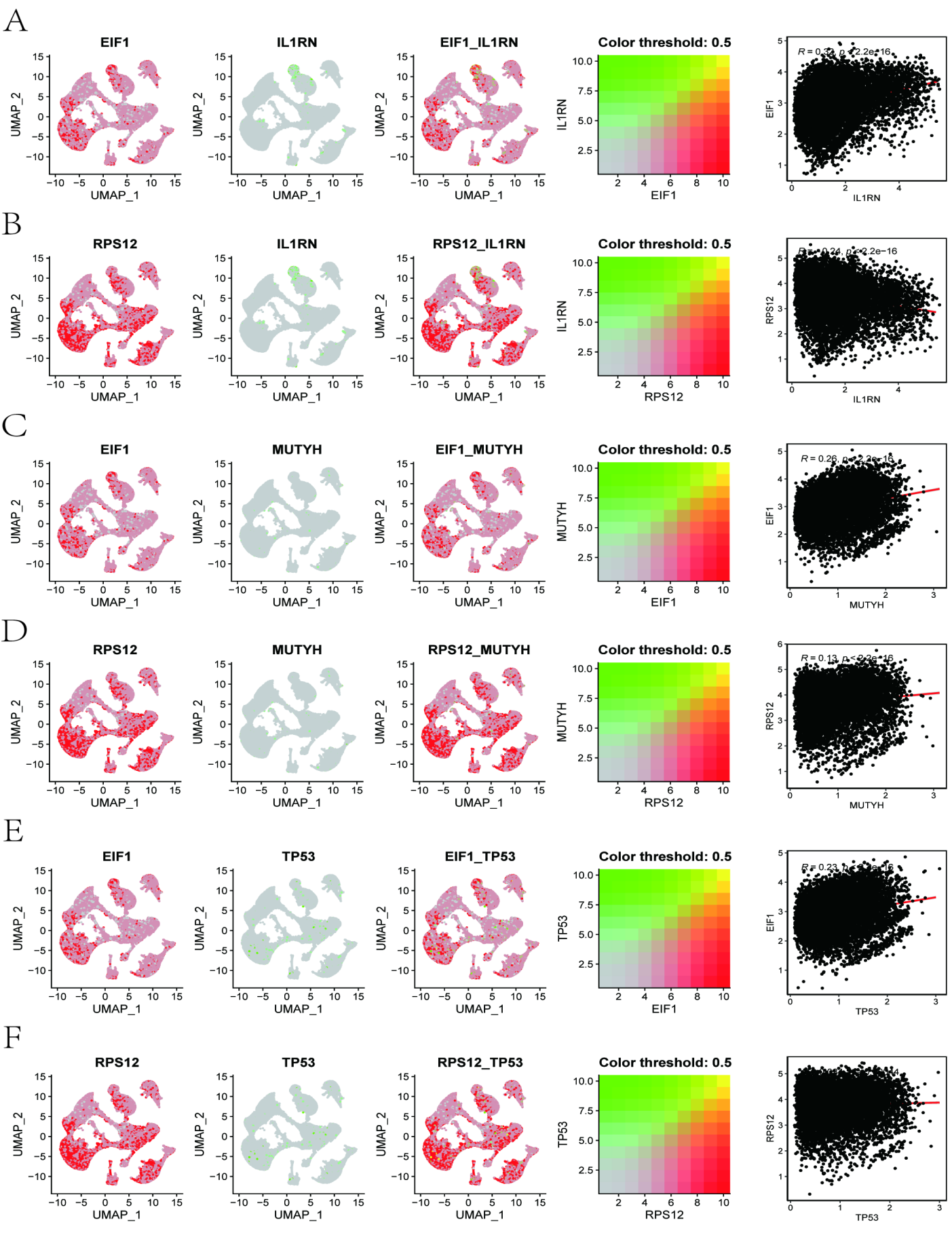


S.2–3. Co-expression of key genes and Top 5 regulatory genes in gastric cancer diseases. Including APC、CHD1、IL-RN、MUTYH、TP53.
